# Interaction of the Amino-Terminal Domain of the ISAV Fusion Protein with a Cognate Cell Receptor

**DOI:** 10.3390/pathogens9060416

**Published:** 2020-05-27

**Authors:** Nicolás Ojeda, Constanza Cárdenas, Sergio Marshall

**Affiliations:** Instituto de Biología, Pontificia Universidad Católica de Valparaíso; Valparaíso 2390302, Chile; nicolas.ojeda@pucv.cl (N.O.); constanza.cardenas@pucv.cl (C.C.)

**Keywords:** ISAV, fusion protein, cell receptor

## Abstract

The infectious salmon anemia virus (ISAV), etiological agent of the disease by the same name, causes major losses to the salmon industry. Classified as a member of the Orthomyxoviridae family, ISAV is characterized by the presence of two surface glycoproteins termed hemagglutinin esterase (HE) and fusion protein (F), both of them directly involved in the initial interaction of the virus with the target cell. HE mediates receptor binding and destruction, while F promotes the fusion process of the viral and cell membranes. The carboxy-terminal end of F (F_2_) possesses canonical structural characteristics of a type I fusion protein, while no functional properties have been proposed for the amino-terminal (F_1_) region. In this report, based on in silico modeling, we propose a tertiary structure for the F_1_ region, which resembles a sialic acid binding domain. Furthermore, using recombinant forms of both HE and F proteins and an in vitro model system, we demonstrate the interaction of F with a cell receptor, the hydrolysis of this receptor by the HE esterase, and a crucial role for F_1_ in the fusion mechanism. Our interpretation is that binding of F to its cell receptor is fundamental for membrane fusion and that the esterase in HE modulates this interaction.

## 1. Introduction

Infectious salmon anemia (ISA) is an aggressive disease that primarily affects Atlantic salmon (*Salmo salar*), one of the most commercially relevant farmed fish. The disease has high mortality rates, with outbreaks occurring at later stages of the breeding process, seriously impacting the industry’s sustainability [1]. ISA has affected all major salmon-producing countries, including Canada, Norway, Scotland, the Faroe Islands, and Chile [2,3,4,5,6].

The etiological agent of ISA is a relatively new virus within the Orthomyxoviridae family and is the only member of the novel *Isavirus* genus [7]. The infectious salmon anemia virus (ISAV) shares similarities with other members of the family, including a segmented single-stranded negative-sense RNA genome and a viral envelope [8,9]. The ISAV envelope contains two major glycoproteins that mediate binding, membrane fusion, and receptor destruction. One of them is a hemagglutinin esterase (HE) which interacts with sialic acids on the susceptible cell surface via the hemagglutinin domain, promoting viral attachment; on the other hand, the esterase acts as a receptor-destroying enzyme (RDE), allowing the release of new viral particles in the context of a productive infection [10,11]. The second ISAV envelope protein is known as fusion protein (F) and mediates the fusion process of the viral and cellular membranes. Cleavage of F by extracellular proteases and a pH-mediated conformational change, once inside the endosome, are necessary for the activation of the fusion mechanism [12,13].

Viral genome segments 6 and 5 code for HE and F, respectively. Virulence markers have been described on both genes, particularly deletions in a highly polymorphic region (HPR) on segment 6, corresponding to a region of the protein located near the transmembrane domain (i.e., stalk region), and insertions near the cleavage site for F in segment 5 [14,15]. Nonvirulent strains of the virus carry a full-length HE gene (HPR0) and have not been isolated in ISAV-permissive cell lines; on the other hand, highly virulent ISAV strains carry versions of the HE gene with deletions in the HPR (HPR∆), suggesting that the HPR0-type gene constitutes the ancestral HE [6,16,17]. Both HPR0 and HPR∆ HE types are fully functional in terms of receptor-binding and receptor-destroying activities, with a specific 5N-4O acetylated sialic acid on the cell surface being identified as the viral receptor [18]. Nevertheless, the HPR length seems to have an influence on the fusion mechanism, with HPR0 HE having lower fusion activity compared to the HPR∆ types [19].

In influenza virus, receptor binding and membrane fusion are mediated by hemagglutinin (HA), with neuraminidase (NA) being responsible for receptor destruction activity [20]. Regarding NA, hydrolysis of sialic acids is not only related to liberation of new viral particles but also to cleavage of mucins from mucus, allowing access to target cells, and migration of virions to appropriated entry sites on polarized cells surfaces [21,22]. Thus, equilibrium in the receptor-binding/receptor-destroying activities has an important role on the initial viral particle interactions with the cell.

In influenza virus, variations in the length of the neuraminidase stalk region have been associated with modifications in tropism and infectivity, with deletions in this region of the NA gene being recognized as virulence markers in avian strains [23,24]. A modification in the length of the NA stalk region may affect the substrate accessibility of the enzyme, altering the receptor-binding/receptor-destroying activities equilibrium and ultimately affecting the viral infectivity [25]. In ISAV, HE contains both receptor-binding and -destroying activities, with no apparent influence from the HPR region over their equilibrium [18].

Interaction of HA with a cellular receptor is a key step for the activation of the fusion mechanism in influenza [26]. In ISAV, no cellular receptor has been described for F, with HE being regarded as solely responsible for viral adhesion to the cell surface [10,27,28]. After proteolytic cleavage, F generates two subunits, linked by disulfide bridges [12]. The carboxy-terminal F_2_, contains structural motifs typically associated to fusion proteins [13]. On the other hand, no function has been suggested for the amino-terminal F_1_. Interestingly, even though the fusion protein from ISAV seems incapable of binding red blood cells (RBCs) from salmon, it does retain its fusion activity in the absence of an HE [12].

Initial interactions of viral particles with the cell are highly important for infection, with fusion of the viral and cellular membranes being a key step. Viral surface proteins and cell glycome diversity are crucial elements regulating virulence and tropism [29,30]. Receptor-binding, receptor-destroying, and membrane fusion activities have a unique distribution in ISAV, compared to other orthomyxovirus; moreover, HE HPR length variation represents a novel mechanism for fusion activity and virulence regulation. In this context, interaction of F with a cell receptor, with regulation of the esterase activity over this receptor via HPR length, represents a feasible explanation for the differential activities described for the HPR0 and HPR∆ fusion systems.

In this study, we aimed to determine the role of the F_1_ domain in the activity of the fusion protein. Due to the lack of a crystal structure, and robust sequence homology to other species, we based our analysis on secondary structure homology and propose a hemagglutinin-like structure for F_1_, suggesting a receptor-binding activity for this domain. We demonstrate the interaction of the F protein of ISAV with a cellular receptor, probably a carbohydrate motif, and the importance of F_1_ for the fusion process. Interestingly, the HE esterase has an effect over the interaction of F with its receptor, which further narrows down the chemical identity of the receptor and suggest a regulatory role for HE not only in the adhesion but also in the fusion mechanism of ISAV. Finally, we propose a model where the stalk length in HE modulates the activity of the viral esterase over the putative F receptor and consequently over the fusion process. These results demonstrate the important role of viral surface proteins in the regulation of infectivity; in particular, they may further define the influence of HPR on the fusion activity in ISAV, suggesting a novel role for F (i.e., receptor interaction). Finally, these results offer new insights into the ISAV fusion system, and the directly related infectivity, aiming to provide a better understanding of the viral behavior.

## 2. Results

### 2.1. Sequence Analysis and Structure Modeling of ISAV F

**Figure 1:**
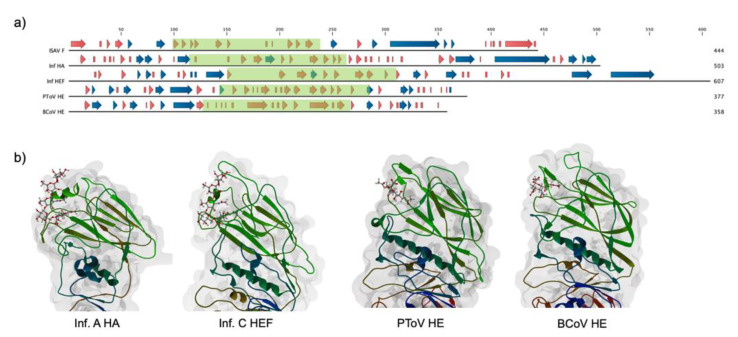
Conservation of secondary structure among viral hemagglutinins. (**a**) Secondary structure comparison for diverse viral hemagglutinins. PSIPRED was used to calculate a secondary structure for infectious salmon anemia virus (ISAV) fusion protein (F) (AAX46273.1), and experimentally defined secondary structures for influenza virus A hemagglutinin (HA) (Inf HA), influenza virus C HEF (Inf HEF), porcine torovirus hemagglutinin esterase (PToV HE), and bovine coronavirus hemagglutinin esterase (BCoV HE) were obtained from their PDB entries (5XRS, 1FLC, 3ILK, and 3CL5, respectively). Beta strands and alpha helices are indicated by red and blue arrows, respectively. Boxed in green are the receptor-binding domains of each protein. There is a clear conservation of beta strands in all of the receptor-binding domains (**b**) Tertiary structure of the receptor-binding domain for the selected viral hemagglutinins. Beta strands constitute a Swiss roll domain, with interstrand loops defining the specificity of interaction in each molecule. The corresponding cellular receptor is shown.

The ISAV F carboxyl-end sequence contains canonical features of a type I fusion protein, and its characteristics have already been thoroughly analyzed [12,31]. Furthermore, the structure of the fusion core has been resolved, allowing for robust definition of the two protein domains: a carboxy-terminal (F_2_), containing the transmembrane domain, a coiled coil region, and a fusion peptide, and an amino-terminal domain (F_1_), of undefined tertiary structure and function [13]. Due to the lack of a crystal structure and robust sequence homology to proteins from other species for the amino-terminal domain of F, we aimed to develop a structural model for this subdomain. A secondary structure analysis of the amino acid sequence for ISAV F revealed a high content of beta strands for the F_1_ subunit, with this topology being characteristic of hemagglutinin domains [32] (Figure 1). Considering that the F_2_ domain resembles the carboxy-terminal domain of influenza virus A HA and influenza virus C hemagglutinin-esterase-fusion HEF, a hemagglutinin-like domain could constitute the amino-terminal structure of the protein.

A putative ligand-binding subdomain (F_1_LB) was defined between positions 100 to 239 of the AAX46273.1 sequence, considering the presence of theoretical alpha helices between residues 84–91 and 249–254 in F_1_, typical of the carboxyl and amino ends of the receptor-binding domain of viral hemagglutinins.

**Figure 2:**
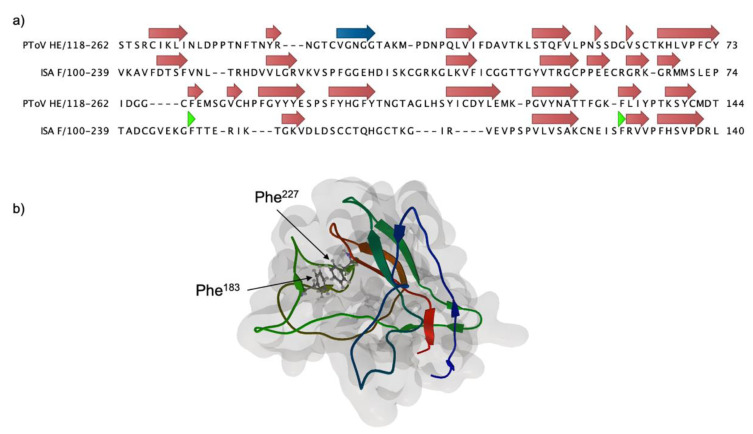
Theoretical model for ISAV F amino-terminal domain (F_1_). (**a**) Secondary structure-based sequence alignment of the PToV HE sequence (3ilk) and the ISAV F_1_ domain. The alignment was used to build a structural model using MODELLER [34]. Beta strands and alpha helices are indicated in red and blue arrows, respectively. The green arrowhead indicates the position of relevant Phe residues (**b**) The theoretical structure for F_1_ displays a conserved Swiss roll conformation. Phe residues at positions 183 and 227, possibly involved in receptor binding, are depicted in ball and stick representation.

A theoretical 3D model for F_1_LB was obtained using comparative modeling using the PToV HE as a template, with a manual alignment based on the secondary structure of each sequence (Figure 2). The ISAV F_1_ beta strands are arranged in an antiparallel fashion, forming a Swiss roll structure, similar to viral hemagglutinins. In canonical hemagglutinins, residues located at the loops connecting the beta strands are involved in ligand binding, with a hydrophobic pocket commonly interacting with the glycan [33]. In our ISAV F_1_ model, two phenylalanine residues are located in this area and thus may play a role in the interaction with a potential receptor.

### 2.2. Lectin Immunofluorescence on ASK Cells Using rHE and rF

**Figure 3:**
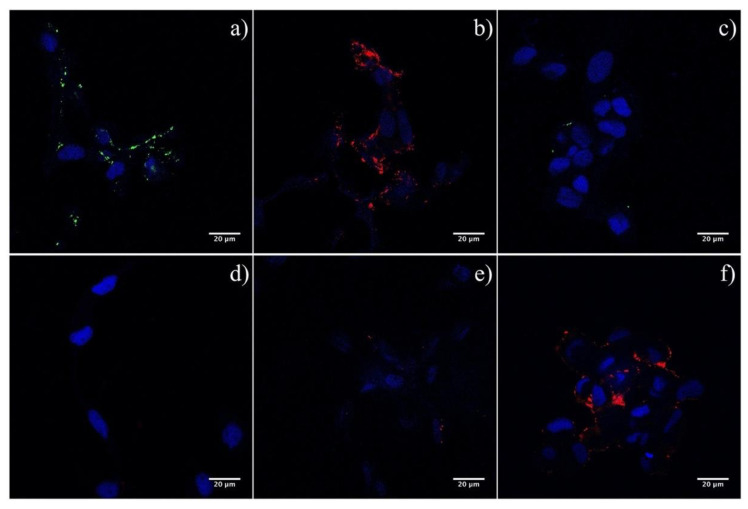
Lectin immunofluorescence on ASK cells using purified 3xFLAG-tagged soluble versions of ISAV HE and F (rHE and rF). (**a**,**b**) Interaction of both viral proteins with a cell receptor was detected using recombinant, soluble, 3xFLAG-tagged versions of ISAV HE and F. Positive reaction of rHE and rF with ASK cells appear in green and red, respectively. (**c**) A total protein extract from sf21 cells was used as control. (**d**) Alkaline treatment of the cells impeded the binding of rF to its receptor. (**e**) Preincubation of ASK cells with rHE blocked the interaction of rF. (**f**) Inactivation of the esterase using DCIC reverted the blockage. Cell nucleus appears stained in blue.

To assess the direct interaction of ISAF F, and HE, with the cell surface, we used purified 3xFLAG-tagged soluble versions of ISAV HE and F (rHE and rF) in lectin immunofluorescence assays, using ASK cells as targets [35]. Indeed, both rHE and rF effectively interacted with the cell surface, as demonstrated by the fluorescent stain pattern (Figure 3a,b). ASK cells pretreatment with NaOH (i.e., saponification) impeded the interaction of rF with the cell, suggesting that the receptor for F may correspond to a sialic acid, as has been already demonstrated for ISAV HE (Figure 3d) [27]. Moreover, preincubation of the cells with soluble HE blocked the adhesion of rF to the cell surface, and chemical pre-inactivation of the esterase on rHE by DCIC treatment eliminated the blockage, suggesting that the initial effect was not due to ligand competition, but rather to the enzymatic activity of rHE (i.e., esterase) over the rF receptor (Figure 3e,f). These results suggest a direct interaction of F with the cell surface and, furthermore, with a sialic acid receptor, considering the results of the saponification and DCIC assays.

### 2.3. Lectin-Mediated Blockage of ISAV Cell Infection, In Vitro

**Figure 4:**
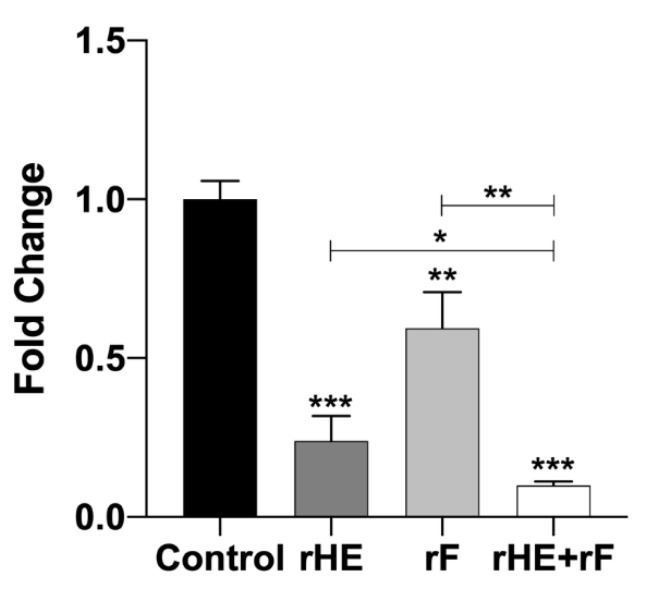
Lectin-mediated blockage of ISAV infection in vitro. ASK cells were preincubated with 50 ng of rHE or/and rF, 30 min prior to infection with ISAV. Forty-eight hours later, inhibition of the infection due to receptor blockage was tested using qRT-PCR to analyze the expression of the viral segment 8 and the housekeeping gene *ELF* in cells. Ct values for segment 8 and *ELF* were used to calculate fold-change for the expression of ISAV relative to the untreated control. Asterisks *, **, and *** indicate statistical significance with *p* < 0.05, *p* < 0.01, and *p* < 0.001, respectively.

To confirm the interaction of HE and F with their cell receptors, ASK cells were pretreated with rHE and/or rF, prior to cell infection. After 48 h, ISAV gene expression was used to evaluate the level of infection compared to mock-treated controls. Figure 4 shows the qRT-PCR inhibitory profiles obtained in each assay. The three treatments had a significant negative effect over the viral infection compared to the untreated control, with a pronounced effect for the rHE treatment (77% inhibition), a lower effect for rF (41.3% inhibition), and the highest inhibitory effect for the treatment with a combination of the two lectins (90.3% inhibition). Notably, there was a significant difference between the double-lectin treatment and each one of the single-lectin treatments, suggesting an additive inhibitory effect on ISAV infection for the rHE/rF combination (Figure 3). These results confirm the interaction of both viral proteins with the cell surface. Moreover, the interaction of each viral protein with a specific receptor is confirmed, which translates in the blockage of infection in this assay.

### 2.4. Membrane Fusion Assays

#### 2.4.1. Importance of F_1_ in the Fusion Mechanism

**Figure 5:**
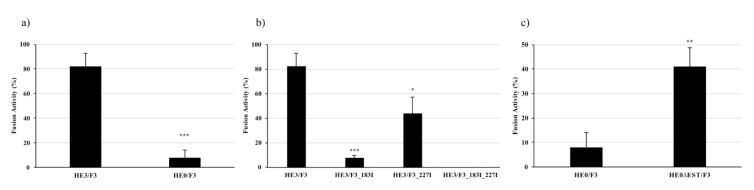
Fusion activity of WT and mutant HE/F combinations. Common bluegill embryo (CHSE/F) cells expressing different combinations of HE and F were fused with R18-labeled salmon red blood cells (RBCs) to measure the fusion activity of each combination, assessing the role of F_1_ and the esterase activity on the process. (**a**) The HE0/F3 system displays significant lower fusion activity than the HE3/F3 combination used as control. (**b**) Mutation of Phe 183 and 227 on F_1_ has a negative effect over the fusion activity, with the double mutation rendering the system completely inactive. (**c**) Esterase inactivation via Ser32Ala mutation on HE0 significantly augments the native fusion activity of the HPR0 system, from 4% to 40%. Asterisks *, **, and *** indicate statistical significance with *p* < 0.05, *p* < 0.01, and *p* < 0.001, respectively.

To assess the role of the F_1_ domain on the fusion activity of F, different ISAV HE and F combinations were evaluated in membrane fusion assays, using transfected CHSE/F cells expressing the viral HE and F proteins and R18-labeled salmon RBCs. After adhesion, cells were subjected to trypsin treatment and low pH to activate the fusion mechanism. Fusion was evident as the transference of fluorescence from the RBC to the CHSE/F cell. We assessed the level of expression of every construct, using immunofluorescence assays, and found effective and comparable expression of all of them (Figures S1–S3). Hemagglutinin esterase and fusion protein from the ISAV HPR3 strain were used as positive controls and initially compared to the HE0/F3 combination. As previously described, HE0 is associated with a less efficient fusion system, with 4% of fusion, compared to the 88% of the positive control (Figure 5a). To evaluate the role of Phe183 and Phe227 from F_1_ in the fusion mechanism, F3 mutants were developed, carrying Phe to Ile mutations (Table 1). Both residues seem to have an important role in the protein functionality, with the Phe183Ile mutation reducing the fusion activity to a 7.1%, the Phe227Ile mutation reducing fusion activity to a 43.7%, and the double mutant completely eliminating the fusion activity (Figure 5b). These results demonstrate an important role for these two residues, and the F_1_ domain, in the protein functionality and further suggest their participation in a receptor-binding site, with direct participation of these two amino acids.

#### 2.4.2. Influence of HE Esterase over the Fusion Mechanism

Considering our results, we hypothesized that the esterase in HE has the capacity to hydrolyze the receptor for F_1_ and that this interaction is crucial for the fusion activity of F. To analyze the influence of the HE HPR0 esterase activity over the fusion mechanism, an HE Ser32Ala mutant was developed, rendering the enzyme inactive [10]. Interestingly, the fusion mechanism with the HE0∆EST/F3 combination showed a level of 40% of fusion activity, 10 times the activity displayed by the HE0/F3 system, implying a negative influence of the enzyme over the viral fusion mechanism (Figure 5c). This result coincides with our hypothesis, suggesting that the esterase in HE has activity against the F receptor and that this activity has a detrimental effect over the membrane fusion mechanism. Moreover, this result led us to the hypothesis that the diminished fusion activity associated with the HE HPR0-type may be connected to the esterase activity of this protein.

## 3. Discussion

The present study demonstrated a crucial role for the F_1_ domain of the ISAV F protein in membrane fusion, a key step in the viral infection process. F_1_ interacts with target cells, and its putative receptor may be hydrolyzed by the esterase present in the HE viral protein. The enzyme has influence over the fusion phenomena, presumably hydrolyzing the F receptor. Finally, we hypothesize that the activity of the enzyme over the F cellular ligand is regulated by the length of the HPR region, where an HE with an extended region (i.e., HPR0) could be more active against this putative sialic acid. Hydrolysis of the F receptor upon viral adhesion may impede F_1_ interaction with the cell, diminishing the membrane fusion process and the subsequent viral infection. These findings represent new data by which the functional basis of the ISAV HPR0 behavior and the virus infection mechanism may be understood.

Interaction with cell receptors is a key element for the activation of viral infective mechanisms, either directly or by conducting the viral particle to cell regions where these systems can be activated [36,37]. On the other hand, receptor destruction plays an important role in the release of the new viral particles. Ultimately, differential expression of receptors in particular cells may regulate the pathogen’s tropism [23]. In the case of ISAV, adhesion to the target cells has been attributed to the viral HE interaction with a sialic acid (5-N, 4-O acetylated) on the cell surface [28]. The membrane fusion activity, on the other hand, is mediated by the so-called fusion protein (F), eventually promoting the transference of the viral content into the cell [12]. Finally, the release of the new virions is related to the enzymatic activity of the esterase in HE, which acquires the receptor-destroying enzyme (RDE) quality [28]. In ISAV, this simplified scheme does not cover the real complexity of the system.

One of the greatest questions surrounding ISAV is the relation between the nonvirulent HPR0 strains and their virulent counterparts. It has been proposed that the former are the ancestral strains, given that they have the genetic potential (i.e., full HE gene) [38]. Certainly, the fact that HPR0 strains have not been isolated in culture has made the study of this potential relation very difficult. The particular mechanisms involved in the lack of virulence and the impossibility of in vitro isolation have not been fully resolved yet.

The molecular characteristic that defines the ISAV HPR0 strains is the presence of an elongated version of the HE gene, compared to the HPR∆ types. Both HPR0 and HPR∆ HEs are fully functional in terms of their receptor-binding activities, as proved by hemadsorption assays using salmon RBC. Interestingly, when the assay was performed using rabbit RBCs, the esterase activity seemed to be more pronounced in the HPR0-type, as manifested in the earlier release of this type of erythrocyte, as compared to a HPR∆ type [18]. This suggests that the activity of the enzyme over the receptor present in the rabbit RBCs may be regulated by the length of the HPR and that this ligand for HE is different from the one located on salmon erythrocytes. On the other hand, the fact that salmon RBCs are not released in the hemadsorption assays suggests that the HPR-esterase regulation may not apply to the activity over the “primary ligand” (5N-4O sialic acids) described as the main receptor for ISAV HE. This stalk-length-mediated regulation of the RDE activity phenomenon may have a parallel in influenza, where the length of the neuraminidase stalk region seems to be related to the viral tropism in avian strains [23,25].

Interestingly, in ISAV, the fusion process is affected by the length of the HPR in HE, with a full stalk being associated to a reduced fusion activity [19]. Interaction of the hemagglutinin (HA) with its receptor in influenza virus is a key step to the activation of the fusion mechanism, before the proteolytic cleavage and endosomal acidification [26]. An analogous condition is true for paramyxovirus, were a structural change is promoted by the interaction of HN with its receptor, which in turn is transmitted to the F protein, triggering a rearrangement which ultimately leads to the membranes fusion [39]. A similar model has been proposed for ISAV, where the direct interaction of HE and F could be responsible for the activation of the latter and the concomitant dissociation upon receptor binding; although this model is robust, no conformational changes were detected on the ISAV HE structure upon receptor binding, and thus, HE is not likely the trigger to activate ISAV F [11,19]. On the other hand, there have been no reports of a direct interaction of the fusion protein of ISAV with the cell, prior to the conformational change and insertion of the fusion peptide.

The equilibrium between the receptor-binding and -destroying activities is a key element for the infective process, not only in terms of the “input/output” ratio of viral particles, but also in the initial interaction of the pathogen with the cell surface [37,40]. In ISAV, if the primary receptor (5N-4O sialic acids) binding via HE is not affected by the HPR length/esterase activity, possibly a secondary viral protein–cellular ligand interaction is regulated by those elements. Considering these findings, we postulated a putative interaction of F, prior to activation, with a cell receptor.

There have been no reports of an experimental structure for the ISAV F_1_ subunit, and protein sequence homology to proteins from other species is very low, which makes it very difficult to project a model structure or function to this domain. Hemagglutinin tertiary structures are relatively conserved among species, even though they share modest sequence homology [32,41]. Furthermore, the F_2_ domain of ISAV F bears structural homology to the fusion domain of influenza virus HA, that is, a coiled coil structure and a hydrophobic fusion peptide, which suggest a conserved structure for the amino-terminal domain as well. Effectively, a secondary structure prediction for the ISAV fusion protein revealed a high content of beta sheets, which is characteristic of hemagglutinin domains, forming a Swiss roll type structure (Figure 1) [42]. Sequence alignment allowed for comparative modeling of a subdomain in F_1_ using the structure of the PToV HE as template (Figure 2). Although there is a low level of amino acid conservation between model and template (38.31% identity), there is structural homology between the proteins, considering the presence of beta sheets in both and the location of particular hydrophobic residues [32]. Typically in hemagglutinins, hydrophobic amino acids located between the beta strands are directly involved in ligand binding [32,42,43,44,45].

Using heterologous expression of ISAV HE and F, we have previously obtained soluble, 3xFLAG-tagged versions of the viral proteins [35]. These were used to perform lectin immunofluorescence assays on ASK cells, a fish cell line conventionally permissive to ISAV infection. The assays revealed a clear interaction of both rHE and rF with a cellular element, possibly a membrane-bound ligand (Figure 3a,b). Interaction of rF with its putative receptor was hampered by preincubation of the cells with rHE, suggesting a competition for the cellular ligand between the two proteins (Figure 3e). Interestingly, treatment of rHE with DCIC, an esterase inhibitor, reverted the effect, suggesting that the enzyme is responsible for the blockage of the interaction of rF with the cell (Figure 3f). ISAV esterase has shown activity against a variety of sialic acids, including 9-O-acetylated sialic acids, which are not necessarily recognized as receptors for HE [28]. These results imply that a sialic acid, acting as receptor for F, may be hydrolyzed by the esterase in HE. Moreover, saponification of the glyco-conjugates present on the ASK cell membrane impeded the interaction of rF with its receptor, confirming the chemical nature of the molecule (Figure 3e).

Interaction of rHE and rF with target cells was further demonstrated by the inhibition of ISAV infection in lectin-treated ASK cells. Incubation of the cells with the soluble lectins, prior to cell infection, blocked the viral receptors on the cell surface, diminishing infection as reflected by a lower expression of the viral genomic segment 8 as compared to the mock-treated controls (Figure 4). Preincubation of the cells with rHE had a more pronounced effect on viral infection inhibition than the rF treatment, this being probably related to the different strength of the interaction of HE and F with their receptors. Thus, the reported lack of hemadsorption capacity on F may be due to a weak interaction with its receptor on RBCs, where the low-strength binding of the sialic acids does not allow the adhesion and retention of the erythrocyte by the F-expressing cell [12]. Moreover, treatment of ASK cells with a combination of both lectins resulted in the highest inhibition of viral gene expression/ISAV infection, with significant differences from the single-lectin-treated cells; accordingly, the result suggests the existence of independent receptors for HE and F, manifested in an additive effect for the inhibition, and confirms the importance of the interaction of both proteins with the cell surface for the infective process.

As previously reported, membrane fusion assays revealed a lower fusion activity in the HPR0-type system, compared to a HPR∆ (Figure 5a) [19]. Similarly, we used this approach to evaluate the role of the F_1_ domain in the fusion mechanism. In particular, the importance of the aforementioned phenylalanine residues in the F_1_LB was demonstrated by the Phe183Ile and Phe227Ile mutations, with the system showing a reduction in activity to 7.1% and 43.7%, respectively, compared to the control (Figure 5b). Moreover, the double mutant abolished all fusion activity, proving the importance of these residues for the mechanism and a putative interaction of F_1_ with a cell receptor. Both Phe residues are highly conserved amongst ISAV F sequences reported in GenBank, with only 4% of them showing a Phe183Leu change (data not shown), which correlates with the importance of their function, as proven by these results.

We suggest an influence of the HPR over the ISAV HE esterase activity, considering the results previously reported for hemadsorption with rabbit RBCs, and that this activity may influence the interaction of F with its cell receptor [18]. Indeed, fusion assays using a Ser32Ala mutated version of HPR0 HE showed an increased fusion activity compared to the WT HPR0 HE, demonstrating a negative influence of the enzymatic activity over the fusion mechanism. For the esterase inactive mutant, membrane fusion activity reached up to 40% (Figure 5c). These results coincide with the rHE-mediated impairment of the rF interaction with the cell and the recovery of such interaction with the DCIC treatment (Figure 3), reinforcing the hypothesis of a direct relation of F with a sialic acid on the cell surface and the importance of this event for the activation of the fusion mechanism.

We hypothesize that the combined roles of HE and F define the fusion activity of the viral particle, where synergistic effects modulate this mechanism, to optimize viral replication. Interestingly, even though salmon RBCs are effectively (and irreversibly) agglutinated by ISAV HE and are capable of endocytosis, effective replication of ISAV on these cells has not been confirmed, and they are regarded as poor viral factories for other piscine viruses [27,46,47,48,49]. In this context, the virus may “select” an appropriate cell target, using a secondary receptor (i.e., an F receptor), independent of HE. Thus, the interaction of HE with its receptor, as proved by hemadsorption and hemagglutination, does not suffice for the correct activation of the membrane fusion mechanism and cell infection. Finally, a particular tropism may be related to the HPR0 behavior, where specific cells on the fish support viral replication for the strain (i.e., with high content or high exposure of the F receptor) at low rate and with no deleterious effect to the host. Further studies should focus on resolving the specific identity of this sialic acid and its presence in particular tissues/cells of the fish.

**Figure 6:**
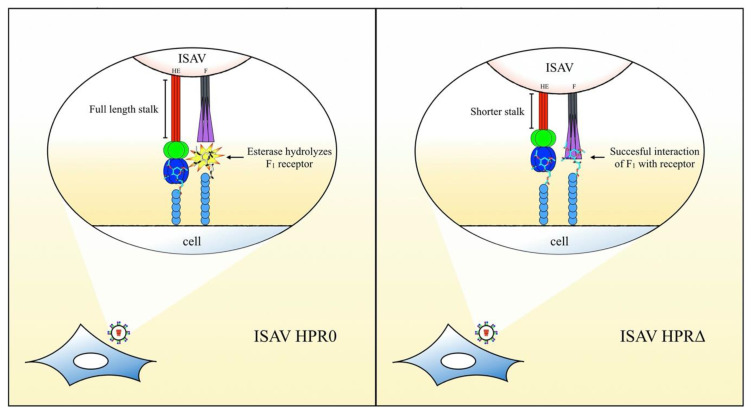
Hypothetical model for the initial interaction of ISAV and roles of the viral surface proteins. The full-length stalk on the HPR0-type augments the activity/accessibility of the esterase over a sialic acid, which acts as a receptor for F. Lack of interaction of F_1_ (in purple) with the cell surface impairs the activation of the fusion mechanism and ultimately diminishes the infective capacity of these strains. On the HPR∆ types, the shortened stalk negatively regulates the enzymatic activity over the F receptor, allowing the interaction of F_1_ with this sialic acid and, finally, the successful activation of the fusion mechanism.

In conclusion, we have demonstrated a novel role for the F protein in ISAV, where interaction of the protein with a sialic acid on the cell surface, prior to activation, can be a key step for the membrane fusion process. Highly conserved amino acids on the F_1_ subdomain are crucial for the protein function and possibly related to ligand binding. Moreover, esterase activity in HE hydrolyses the ligand for F, with the enzymatic activity being regulated by the length of the HPR. Figure 6 depicts the hypothetical model we propose for initial ISAV interaction with the cell surface, derived from our results and their interpretation.

## 4. Materials and Methods

### 4.1. Fish Cell and ISAV Cultures

Atlantic salmon kidney (ASK) cells (ATCC CRL2747) [50] and common bluegill embryo (CHSE/F) cells (formerly known as CHSE-214, ATCC 1681) [51] were cultured in Leibovitz’s L-15 medium with 4 mM glutamine (Gibco) and supplemented with 200 U/mL penicillin, 200 µg/mL streptomycin, 0.5 µg/mL amphotericin, and 10% fetal bovine serum (Gibco), at 20 °C. Cells were grown to 80% confluence and accordingly subcultured.

Field isolates of ISAV corresponding to the HPR3 and HPR7b types were obtained from the Laboratorio de Genética e Inmunología molecular strain collection. Viral infection and propagation were performed using ASK cells, where an 80% confluent cell monolayer was washed twice with L-15 medium and further covered with a viral dilution prepared in L-15 medium. After 4 h of incubation, the viral inoculum was removed, and the cells were washed twice with L-15 medium and further cultured in L-15 medium supplemented with 2% fetal bovine serum and antibiotics, at 17 °C. After 7 days, the cell supernatant was recovered and filtered (0.45 μm), and viral aliquots were collected and stored at −80 °C. A plaque assay was used for virus tittering 12 days post-infection (d.p.i.), as previously described [52].

### 4.2. Spodoptera frugiperda (Sf21) Cells and Baculovirus

*Spodoptera frugiperda* (Sf21) cells were used for recombinant baculovirus production and recombinant protein expression. Cells were grown in suspension culture using SF-900 III medium (Gibco) supplemented with 1% fetal bovine serum, at 28 °C. Suspension cells were seeded at 0.3 × 10^6^ cells/mL, agitated at 150 rpm in 500 mL flasks, and subcultured when reaching (8–9) × 10^6^ cells/mL.

### 4.3. Baculovirus Expression System and Protein Purification

Recombinant baculovirus were developed for the expression and purification of the extracellular domains of ISAV HE and F proteins, using the baculovirus/Sf21 cells expression system, as previously described by Ojeda et al. [35]. For recombinant protein production, a suspension culture of 300 mL of Sf21 cells at 6 × 10^6^/mL was infected using a multiplicity of infection (MOI) of 10. After 120 h culture at 23 °C, Sf21 cells were pelleted at 500× *g*, washed twice with Grace’s Insect Medium, and then suspended in lysis buffer (50 mM Tris-HCl pH 7.4, 150 mM NaCl, 1mM EDTA, and 1% Triton X-100). After a 2 h incubation on ice, samples were sonicated using 20% of amplitude in five 30 s repetitions, with 30 s of cooling between each repetition. Samples were then centrifuged at 12,500× *g* for 30 min to remove cell debris. The supernatant, containing the recombinant proteins, was filtered (0.45 μm) using a low-protein-binding filter (Millipore, Burlington, USA). Recombinant hemagglutinin esterase and fusion proteins (rHE and rF) were purified using the anti-FLAG M2 Magnetic Beads System (Sigma-Aldrich, St. Louis, Missouri, USA) and the Magnetight Separator Stand (Novagen, Darmstadt, Germany) according to the manufacturer’s instructions. Protein concentration for each sample was measured using the BCA Assay Kit and BSA standard (Pierce, Waltham, MA, USA). Approximately 20 µg of each protein was obtained from the infected Sf21 cells.

### 4.4. Lectin Immunofluorescence

ASK cells were seeded on 35 mm^2^ glass bottom dishes (2 × 10^5^ cells). Following a 12 h incubation, the cells were fixed with fresh 4% paraformaldehyde in phosphate-buffered saline (PBS) for 15 min. Then, cells were washed twice with PBS and treated with a blocking buffer (1% BSA and 0.3% Triton X-100 in PBS) for 30 min. After the buffer was removed, 50 ng of rHE or rF, diluted in blocking buffer, was added to the cells, followed by incubation for 1 h. To analyze the influence of HE over the interaction of F with a cell receptor, rHE was pretreated with 10 µM DCIC (Sigma-Aldrich, St. Louis, Missouri, USA) for 30 min before incubating the cells with the recombinant lectin for 1 h [53]. After three PBS washes, 50 ng of rF were added and incubated as described above; a DMSO-treated aliquot of rHE was used as control. ASK cells were treated with 0.1 M NaOH for 30 min for saponification. This procedure results in the de-*O*-acetylation of sialic acids, prior to the lectin incubation step, to evaluate the interaction of rF with sialic acids on the cell [27]. As a negative control, ASK cells were incubated with a protein extract from Sf21 cells (1 µg of total protein). After the lectin incubation steps, cells were washed three times with PBS. Cells treated with single lectins were incubated with an anti-FLAG M2 antibody (Sigma-Aldrich, St. Louis, Missouri, USA) at a 1/100 dilution in blocking buffer. Cells treated with rHE and rF were incubated with an anti-ISAV F (8B2/A4) (Bioschile, Santiago, Chile) at a 1/250 dilution. After 1 h of incubation and three PBS washes, Alexa Fluor 568 conjugated goat anti-mouse (Thermo-Fisher, Waltham, MA, USA) was added to the cells at a 1/200 dilution in blocking buffer. Following a final 1 h incubation, cells were washed three times with PBS, and cell nuclei were stained with a solution of TO-PRO-3 (1 µM in PBS) (Thermo-Fisher, Waltham, MA, USA) for 1 min. After three final washes with PBS, 1 mL of the same buffer was added, and cells were examined by confocal microscopy using the TCSSP5 II confocal microscope (Leica Microsystems Inc., Wetzlar, Germany).

### 4.5. Lectin-Mediated ISAV Infection Inhibition

ASK cells at 80% confluence, growing on a six-well plate (2.5 × 10^5^ cells per well), were washed two times with L-15 medium. Then, each well was incubated with 100 ng of Sf21-expressed rHE, rF, or a mixture of both in L-15 medium. As control, cells were treated with 100 ng of total protein from Sf21 cells. After a 30 min incubation, cells were washed six times with L-15 medium. Then, cells were infected with ISAV HPR7b at an MOI of 0.01, as described above. Total RNA was extracted from the cells 48 h post-infection (h.p.i.) using the TRIzol reagent (Life Technologies, Carlsbad, California, USA) and suspended in nuclease-free water. A 2 μL RNA sample was used as template for qRT-PCR reactions using the Stratagene qRT-PCR III Master Mix according to the manufacturer’s instructions. Specific primers were used to analyze the expression of viral genes (viral segment 8) [54] and that of a cellular housekeeping gene (elongation factor 1α) used for normalization [55]. The fold-change of viral gene expression relative to the control was assessed using the 2^-ΔΔCT^ method, as previously described [56] (Table 2).

### 4.6. Sequence Analysis and Modeling

A reference sequence (AAX46273.1) for the ISAV fusion protein was retrieved from NCBI protein sequence database [15] and was analyzed with the PSIPRED method v3.3 to predict secondary structure [57]. Comparison with the sequences and experimental secondary structures for hemagglutinins from porcine torovirus (PToV) (3i1k), bovine coronavirus (3cl5) and influenza C virus (1flc) was performed, in order to define a putative ligand interacting domain for ISAV F [32,42,45]. A 3D model of a partial F_1_ domain was obtained based on manual alignment with the PToV HE structure sequence. Comparative modeling was performed using the MODELLER interface in UCSF Chimera [34,58], using the PToV HE structure as template. Sequence alignment was performed using Jalview [59]. Alignment and structural representations were obtained using CLC Genomic Workbench (Qiagen, Hilden, Germany).

### 4.7. Molecular Cloning and Expression Vector Construction

Total RNA was extracted from ASK cells infected with ISAV HPR3, as described before, using the TRIzol reagent (Life Technologies, Carlsbad, California, USA) according to the manufacturer’s instructions. A 10 mg kidney sample from an ISAV HPR0 infected salmon was obtained from the Laboratorio de Patógenos Acuícolas, and total RNA was extracted using the TRIzol Reagent, as described above. cDNA was synthesized from 5 μg of total RNA with M-MLV Reverse Transcriptase (Invitrogen, Carlsbad, California, USA) using 500 ng of oligo (dT)_12-18_, 1 mM of each dNTP, and 200 U of M-MLV Reverse Transcriptase according to the manufacturer’s instructions. The ISAV HPR3 HE and F genes (HE3 and F3, respectively) and the ISAV HPR0 HE gene (HE0) were amplified through polymerase chain reaction (PCR) using the corresponding cDNA as template and cloned in an appropriate expression vector. In detail, to analyze the influence of the esterase in HPR0 HE and the influence of amino acids Phe 189 and Phe 227 in F_1_ over the fusion process, point mutations were added to the open reading frames (ORFs), using overlap extension PCR (OE-PCR) [60]. The ORFs for eGFP and mCherry were added to the 3′ end of the HE and F ORFs, respectively, to obtain fusion proteins and directly evaluate the construct’s expression. An overview of the different constructs is presented in Table 1. The wild type (WT), mutated, and fusion ORFs were cloned in the p3xFLAG-CMV14 vector (Sigma-Aldrich, St. Louis, Missouri, USA). Accordingly, primers were designed for each process (Table 2). The PCR reactions were performed in a 20 µL total volume using a 1 µL cDNA sample as template, with 1X HF Phusion Buffer (NEB, Ipswich, MA, USA), 200 µM dNTPs, 500 nM of each primer, 1 M betaine, and 1 U of Phusion DNA Polymerase (NEB, Ipswich, MA, USA). Thermal conditions used for PCR reactions were as follows: 3 min at 95 °C, followed by 40 cycles of 30 s at 93 °C, 30 s at 55 °C, and 2 min at 72 °C, with a final extension stage of 5 min at 72 °C. PCR products were cloned in the pJET 1.2/Blunt vector using the CloneJET PCR Cloning Kit (Thermo Fisher, Waltham, MA, USA) according to the manufacturer’s instructions. After digestion with specific restriction enzymes (Promega, Madison, Wisconsin, USA) and gel electrophoresis, gene fragments and the linearized p3xFLAG-CMV14 vector were purified with the GeneJET Gel Extraction Kit (Thermo Fisher, Waltham, MA, USA). For cloning in the p3xFLAG-CMV14 vector, a mixture containing 10 ng of the digested expression vector and 100 ng of the digested HE or F gene inserts were ligated using the Rapid DNA Ligation Kit (Thermo Fisher, Waltham, MA, USA) according to the manufacturer’s instructions. Recombinant plasmids (pDNA) were isolated using the GeneJET Plasmid DNA Kit (Thermo Fisher, Waltham, MA, USA) from transformed DH5α cells grown in LB broth. The cloned sequences and expression constructs were verified by sequencing (Macrogen, Seoul, Korea) using the cmv30F and cmv24R primers.

### 4.8. Cell Transfection

Exponentially growing CHSE/F cells were seeded on glass bottom 35 mm^2^ dishes and cultured overnight, as described above. Cells were washed twice with PBS and then incubated in L-15 medium. A transfection mixture was prepared for each dish, diluting transfection reagent FuGENE (Promega, Madison, Wisconsin, USA) in 100 µL of L-15 medium and then adding pDNA of the HE and/or F expression constructs. Here, 250 ng of the HE constructs and/or 500 ng of the F constructs were added to the mixture [12]. FuGENE was used in a reagent:pDNA (µL:µg) ratio of 3:1. After vortexing, the mixture was incubated for 30 min and then added to the cells dropwise. After a 4 h incubation at 20 °C, the medium was removed and the cells washed three times with L-15 medium. Cells were then incubated in supplemented L-15 medium for 48 h, as described above, to evaluate protein expression and perform functional analyses.

### 4.9. Heterologous Protein Expression Analysis

To evaluate the level of expression of the WT and mutant HE and F, immunofluorescence assays were performed on transfected CHSE/F cells, using the anti-FLAG M2 antibody to detect the 3pFLAG tag on the 3′ end of each construct. After two PBS washes, cells were fixed with 4% paraformaldehyde and then treated with blocking buffer as described above. Anti-FLAG M2 antibody was added to the cells at a 1/100 dilution in blocking buffer. After 1 h incubation and three PBS washes, Alexa Fluor 568 conjugated goat anti-mouse was added to the cells at a 1/200 dilution in blocking buffer. After a 1 h incubation, cells were washed three times with PBS, adding a final 1 mL of the same buffer, and then five representative fields of each transfected dish were analyzed. Images were captured using the TCSSP5 II confocal microscope and then analyzed using the Analyze Tool of the FIJI software package. Corrected total cell fluorescence (CTCF) was calculated using CTCF = integrated density ‒ (selected area × background fluorescence) and used as a measure of fluorescence intensity and protein expression level [61]. In parallel, the expression of the HE-eGFP and F-mCherry fusion constructs was evaluated on the co-transfected cells to ensure the co-expression of both proteins. As described above, five fields of each transfected dish were evaluated by direct observation on the confocal microscope.

### 4.10. Hemadsorption and Membrane Fusion Assays

Blood samples (500 µL) were obtained from pre-smolt *Salmo salar* (40 g) after appropriate sedation in 0.001% benzocaine (Centrovet, Santiago, Chile) for 10 min. Syringes and collection tubes were treated with an anticoagulant solution containing 10 mM sodium citrate, prior to blood collection. Purified red blood cells (RBC) were obtained via sedimentation of blood cells after centrifugation at 110× *g* for 10 min. Cells were washed three times with PBS and finally re-suspended in the same buffer to obtain a 0.5% RBC suspension.

For the hemadsorption assays, 1 mL of a 0.05% RBC suspension in PBS was added to CHSE/F cells expressing WT HE or their mutants. Cells were incubated for 1 h and then washed six times with L-15 medium to remove unbound cells. Bound erythrocytes were permeabilized by adding a solution of 0.05 M NH_4_Cl, followed by incubation for 1 h; the hemoglobin-containing supernatant was then transferred to a 96-well plate for absorbance reading at 540 nm to measure hemadsorption [18].

For the membrane fusion assays, RBCs membranes were fluorescently labeled with octadecyl rhodamine B chloride (R18) (Molecular Probes, Eugene, Oregon, USA). Briefly, 15 µL of a 1 mg/mL R18 solution was quickly added to 10 mL of a 1% RBC suspension in PBS. The mix was incubated for 15 min at 120 rpm agitation. Then, 30 mL of supplemented L-15 medium was added, incubating for an additional 20 min. Erythrocytes were sedimented by centrifugation at 110× *g* and washed six times with 50 mL of PBS to remove the excess of dye. RBCs were re-suspended in PBS to obtain a 0.1% suspension [62].

To evaluate the fusion activity of the different combinations of ISAV surface proteins, CHSE/F cells expressing WT or mutant HE and F were washed two times with L-15 medium and then incubated with 20 µg/mL trypsin for 15 min. The enzyme was then inactivated by 10 min incubation with supplemented L-15 medium. Cells were washed three times with L-15, and then 1 mL of the R18-labeled RBCs was added. After 15 min, erythrocytes were removed, and the cells washed six times with L-15 medium. The fusion mechanism was activated by adding 1 mL of pH 4.5 adjusted L-15 medium and incubating for 30 min. The acid medium was removed and cells were fixed with 4% paraformaldehyde, as described above, after three washes with L-15 medium. Cells were then analyzed using the confocal microscope, measuring the fusion activity as the percentage of membrane-fused cells (i.e., where fluorescence was transferred from the bound RBC to the CHSE/F cell) relative to the total number of CHSE/F cells with bound erythrocytes. Ten fields of each dish, with comparable levels of bound RBCs, were analyzed [63].

### 4.11. Statistical Analysis

The results are presented as the means ± standard deviations of triplicate determinations. Statistical significance of the data was determined by using a Student’s *t*-test. In all figures, *p*-values <0.05, <0.01 and <0.001 are indicated by *, ** and ***, respectively, and were considered significant as properly indicated.

## Figures and Tables

**Table 1:** Heterologous expression constructs developed to evaluate the fusion mechanism of ISAV. The combination of HE and F from an HPR3 strain (HE3 and F3, respectively) was used as positive control. A combination of HE from HPR0 (HE0) and F3 was used to evaluate the influence of the stalk length on the system. Mutants were designed to determine the influence of the F_1_ domain in F, and the esterase activity, accordingly. All wild type (WT) and mutant open reading frames (ORFs) were cloned in the p3xFLAG-CMV14 expression vector. All constructs were 3xFLAG-tagged at the carboxy-terminal end. Fluorescent fusion proteins were developed for each construct, adding eGFP or mCherry to the carboxy-terminal end of the HEs and Fs, respectively (not shown in table).

Construct Name	Description	Comment
HE3	HE from HPR3 strain	Influence of HPR length in the fusion process
F3	F from HPR3 strain	
HE0	HE from HPR0 sample	
F3_183I	F3 with Phe183Ile mutation	Influence of F_1_ in the fusion process
F3_227I	F3 with Phe227Ile mutations	
F3_183I_227I	F3 with double mutation: Phe183Ile and Phe227Ile	
HE0∆EST	HE0 with Ser32Ala mutation: inactive esterase	Influence of the HE0 esterase activity in the fusion process

**Table 2:** Primers used to amplify the viral ORF corresponding to HE and F proteins, to add restriction sites for cloning in p3xFLAG-CMV14, to mutate specific residues in both, and to evaluate the expression of viral and cellular genes. HE was cloned between the Not I and Kpn I sites on the expression vector. For the HE-eGFP fusion, an Xba I site was added to the 3′ and 5′ sites of HE and eGFP, respectively, due to a Kpn I site present in the fluorescent protein gene. HE and F fusion proteins were cloned between the Not I/Bam HI and Eco RI/Bam HI sites of the p3xFLAG-CMV14 vector.

Primer Name	Sequence	Observations
**ORF Amplification**		
5HE2F	ATGGCACGATTCATAATTTTATTC	HE ORF amplification
3HE2R	TTAAGCAACAGACAGGCTC	HE ORF amplification
5F2F	ATGGCTTTTCTAACAATTTTAG	F ORF amplification
3F2R	TTATCTCCTAATGCATCCC	F ORF amplification
**Cloning**		
5HENotF	GCGGCCGCGATGGCACGAT	Adds Not I site to 5′ end of HE ORF
3HEKpnIR	GGTACCGACAGGCTCG	Adds Kpn I site to 3′ end of HE ORF
HE3XbaI	TCTAGAAGACAGGCTCG	Adds Xba I site to 3′ end of HE ORF
5FEcoRI	GAATTCTATGGCTTTTCTAAC	Adds Eco RI site to 5′ end of F ORF
3FKpn1R	GGTACCGATCTCCTAATGC	Adds Kpn I site to 3′ end of F ORF
GFP5XbaI	TCTAGAATGGTGAGCAAGG	Adds Xba I site to 5′ and of eGFP ORF
RGFPBam	GGATCCTTACTTGTACAGCTC	Adds Bam HI site to 3′ end of eGFP ORF
cherryKpnF	GGTACCCATGGTGAGCAAGG	Adds Kpn I site to 5′ end of mCherry ORF
cherryBamR	GGATCCCTACTTGTACAGCTC	Adds Bam HI site to 3′ end of F ORF
**Mutations**		
HESdAFwd	CCTGGTTAGGTGACGCTCGAAGCG	Ser32Ala mutation in HE
HESdARev	TGGACTGATCGCTTCGAGCGTCACC	Ser32Ala mutation in HE
183Fwd	GGAGTGGAAAAAGGCATTAC	Phe183Ile mutation in F
183Rev	AATCCTTTCCGTTGTAATGCC	Phe183Ile mutation in F
227Fwd	TGCAATGAAATTTCAATCAGAG	Phe227Ile mutation in F
227Rev	GAACGGCACTACTCTGATTG	Phe227Ile mutation in F
**qPCR**		
Seg8Fwd	CTACACAGCAGGATGCAGATGT	Viral segment 8 evaluation
Seg8Rev	CAGGATGCCGGAAGTCGAT	
Seg8Probe	FAM-CATCGTCGCTGCAGTTC-TAMRA	
ELF1Fwd	CCCCTCCAGGACGTTTACAAA	Cellular *ELF1* evaluation
ELF1Rev	CACACGGCCCACAGGTACA	
ELF1Probe	FAM-ATCGGTGGTATTGGAAC-TAMRA	

## Supplementary Materials

The following are available online at https://www.mdpi.com/2076-0817/9/6/416/s1, Figure S1: Immunofluorescence assays and heterologous protein expression levels in CHSE/F cells. Transfected cells were evaluated 48 h post-transfection via immunofluorescence using the ANTIFLAG M2 antibody. Proteins where detected in vesicles and membranous compartments of the cell, in accordance with their membrane-bound nature. All proteins had comparable levels of expression, as determined by fluorescence intensity measurements. Hemagglutinin-esterase and Fusion Protein constructs appears in green and red, respectively; Figure S2: Co-expression of HE and F in transfected CHSE/F cells. Fusion proteins for HE and F where developed to assess the correct co-expression of proteins on co-transfected cells. As described above, both HE and F where located at vesicles and membranous compartments of the cell, in accordance with their membrane-bound nature; Figure S3: Hemadsorption levels for CHSE/F expressed HEs. Forty-eight hours post transfection, salmon RBCs were added to CHSE/F cells and hemadsorption levels were assessed measuring the hemoglobin released after erythrocytes were permeabilized, via spectrophotometry. The graph shows the absorbance at 540 nm for triplicate samples. There was no significant difference between the HE constructs.
