# Supplementary material for: Interaction of the Amino-Terminal Domain of the ISAV Fusion Protein with a Cognate Cell Receptor
Source: Pathogens. 2020 May 27;9(6):416. doi: 10.3390/pathogens9060416 (PMC7350309; doi:10.3390/pathogens9060416)
Supplement: Supplementary file 1 [file pathogens-09-00416-s001.pdf]

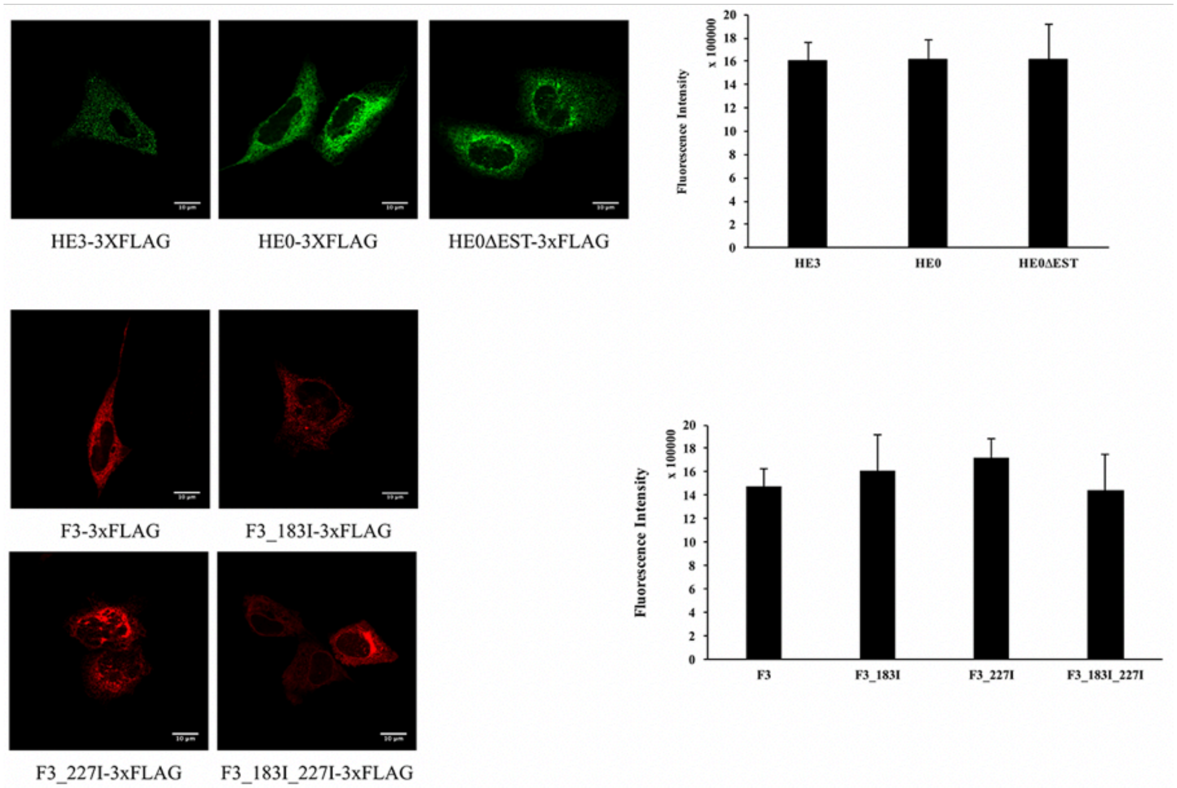

Figure S1: Immunofluorescence assays and heterologous protein expression levels in CHSE/F cells. Transfected cells were evaluated 48 hours post-transfection via immunofluorescence using the ANTI-FLAG M2 antibody. Proteins where detected in vesicles and membranous compartments of the cell, in accordance with their membrane-bound nature. All proteins had comparable levels of expression, as determined by fluorescence intensity measurements. Hemagglutinin-esterase and Fusion Protein constructs appears in green and red, respectively.

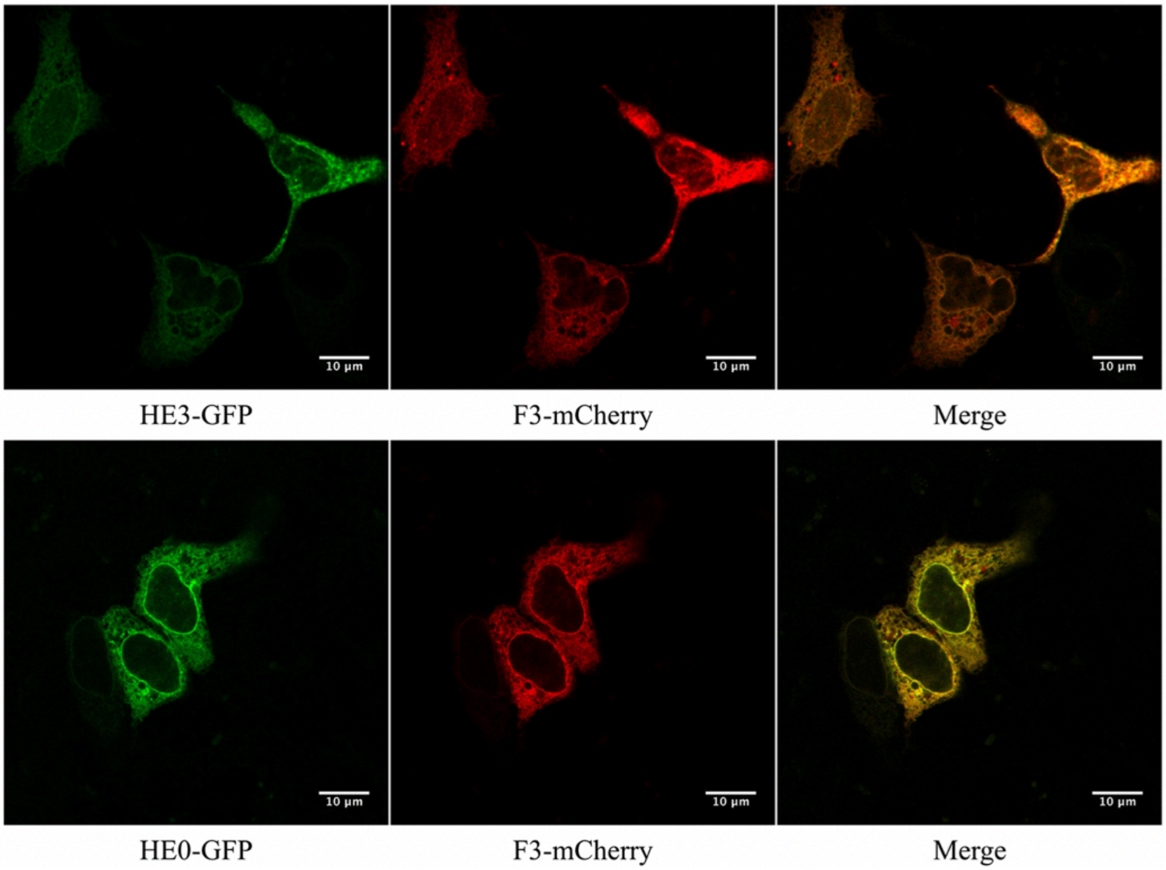

Figure S2: Co-expression of HE and F in transfected CHSE/F cells. Fusion proteins for HE and F where developed to assess the correct co-expression of proteins on co-transfected cells. As described above, both HE and F where located at vesicles and membranous compartments of the cell, in accordance with their membrane-bound nature.

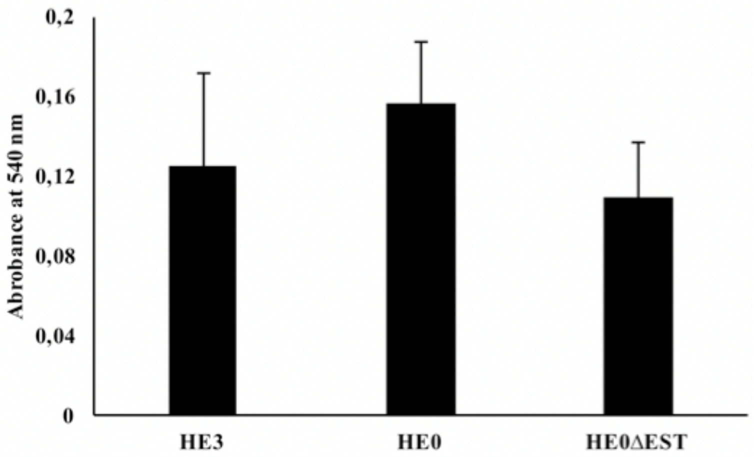

Figure S3: Hemadsorption levels for CHSE/F expressed HEs. Forty-eight hours post transfection, salmon RBCs were added to CHSE/F cells and hemadsorption levels were assessed measuring the hemoglobin released after erythrocytes were permeabilized, via spectrophotometry. The graph shows the absorbance at 540 nm for triplicate samples. There was no significant difference between the HE constructs.
